# Supplementary material for: Aberrant choroid plexus formation drives the development of treatment-related brain toxicity
Source: Commun Biol. 2025 Feb 22;8:276. doi: 10.1038/s42003-025-07736-2 (PMC11846864; doi:10.1038/s42003-025-07736-2)
Supplement: Supplementary file 1 — Supplementary material [file 42003_2025_7736_MOESM1_ESM.pdf]

Supplementary text and figures

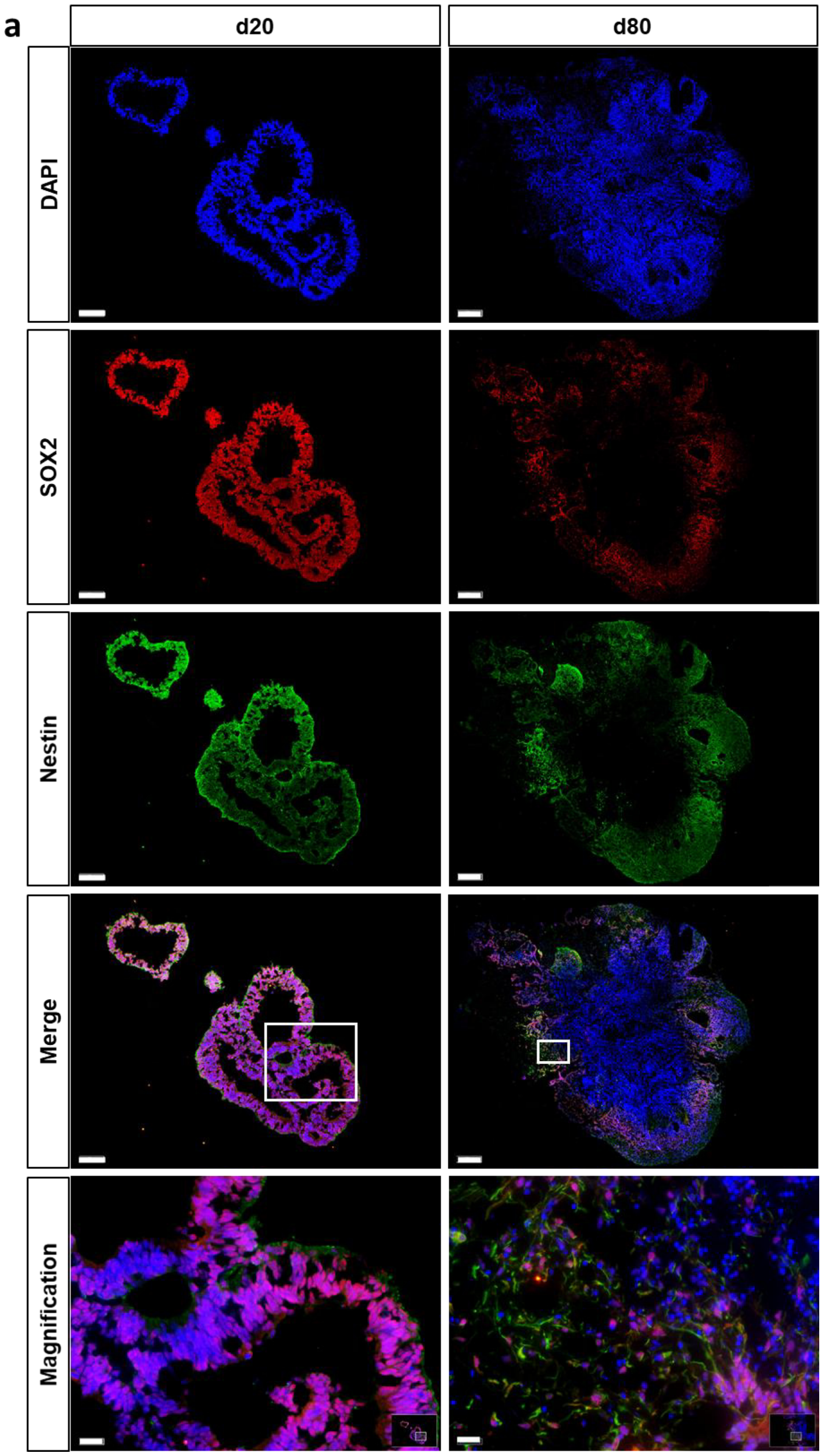

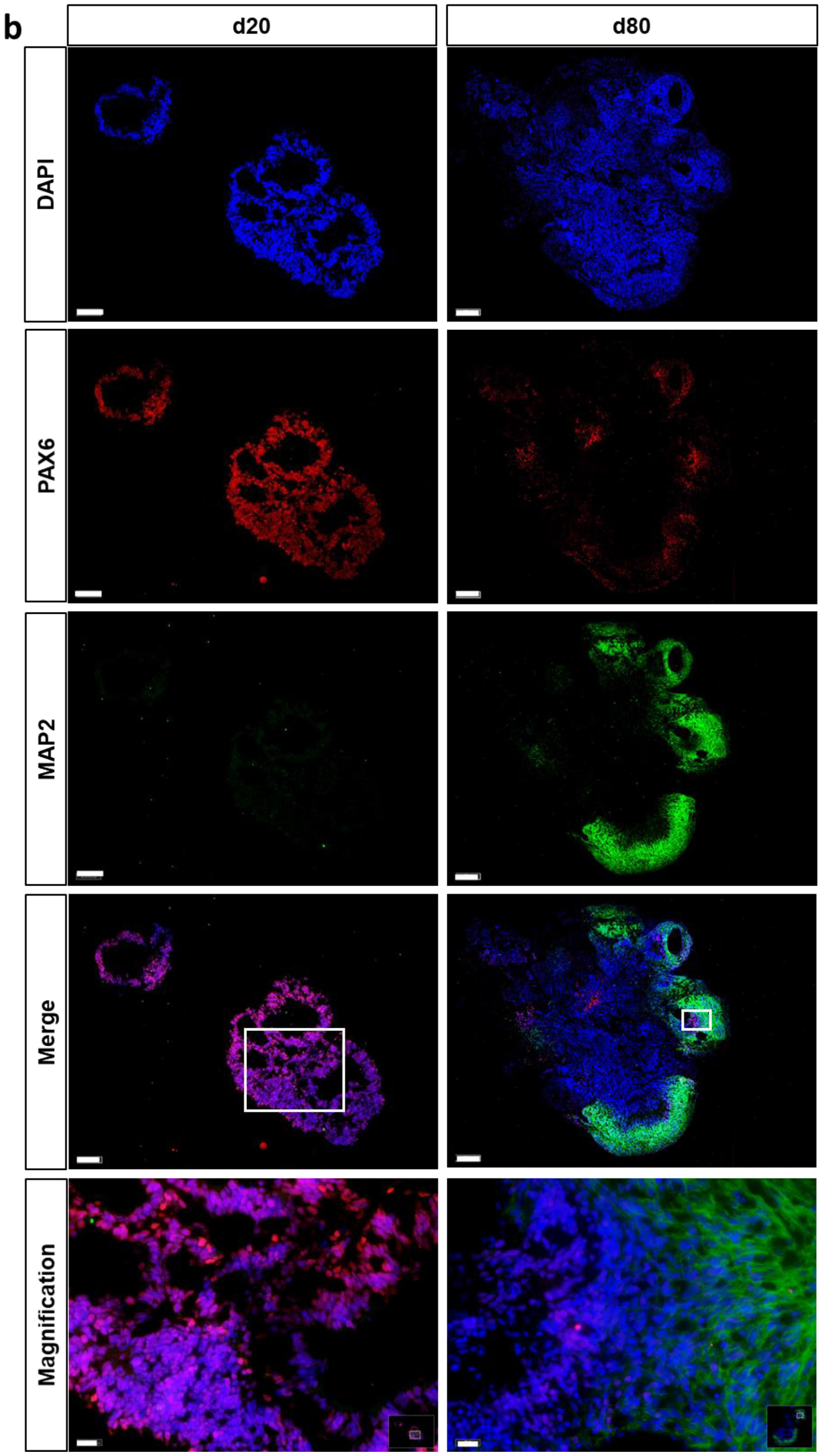

Figure S1. Differentiation and characteristics of organoids at d20 (immature) and d80 (mature). a Representative immunofluorescence staining of SOX2 (red) and nestin (green) in d20 and d80 cerebral organoids, nuclei stained with DAPI (blue), scale bar: 200 µm, and 20 µm for magnification. **b** Representative immunofluorescence staining of PAX6 (red) and MAP2 (green) in d20 and d80 cerebral organoids, nuclei stained with DAPI (blue), scale bar: 200 µm, and 20 µm for magnification.

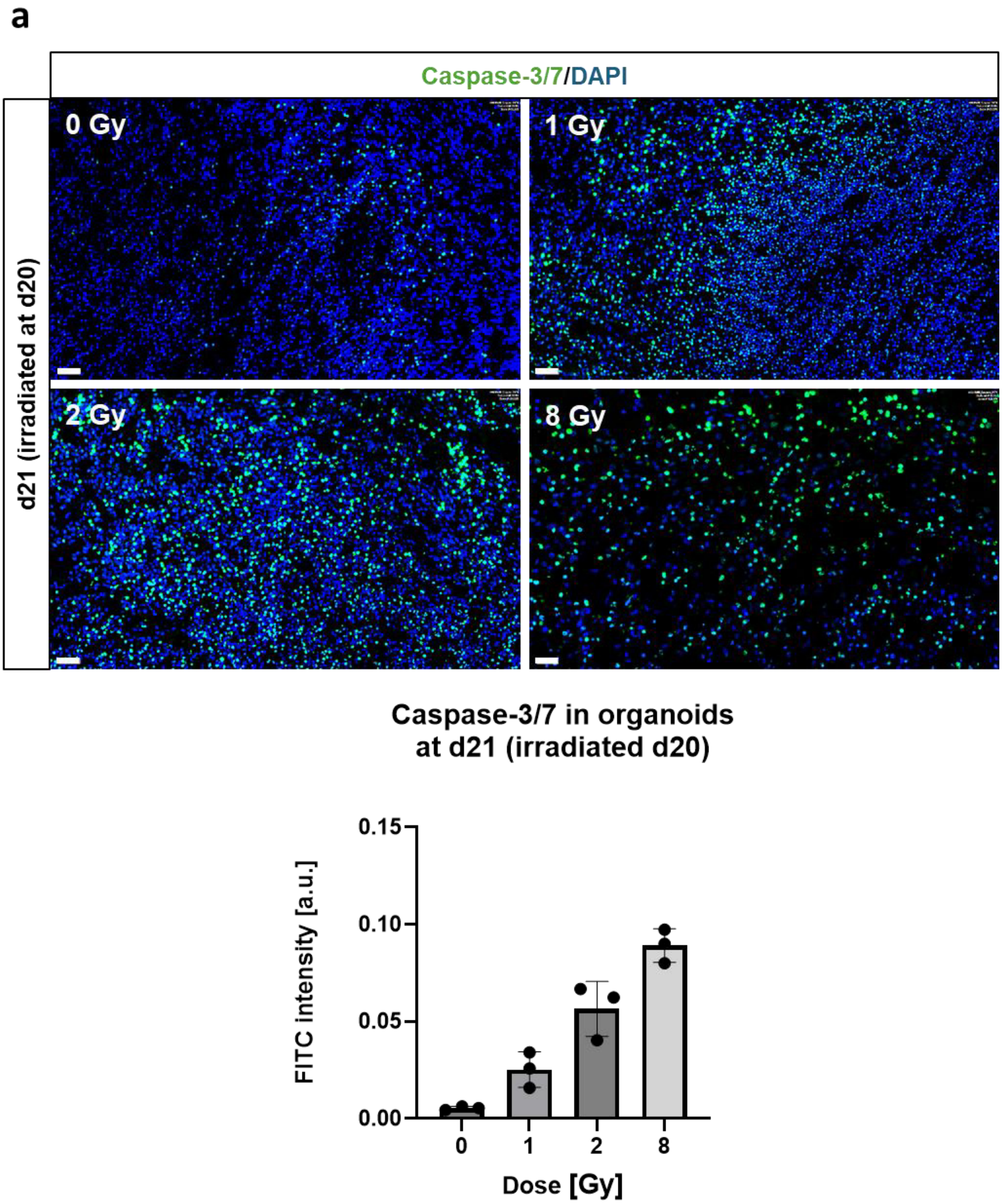
a
Caspase-3/7/DAPI
d21 (irradiated at d20)
0 Gy
1 Gy
2 Gy
8 Gy
Caspase-3/7 in organoids
at d21 (irradiated d20)
FITC intensity [a.u.]
0.15
0.10
0.05
0.00
0
1
2
8
Dose [Gy]

b

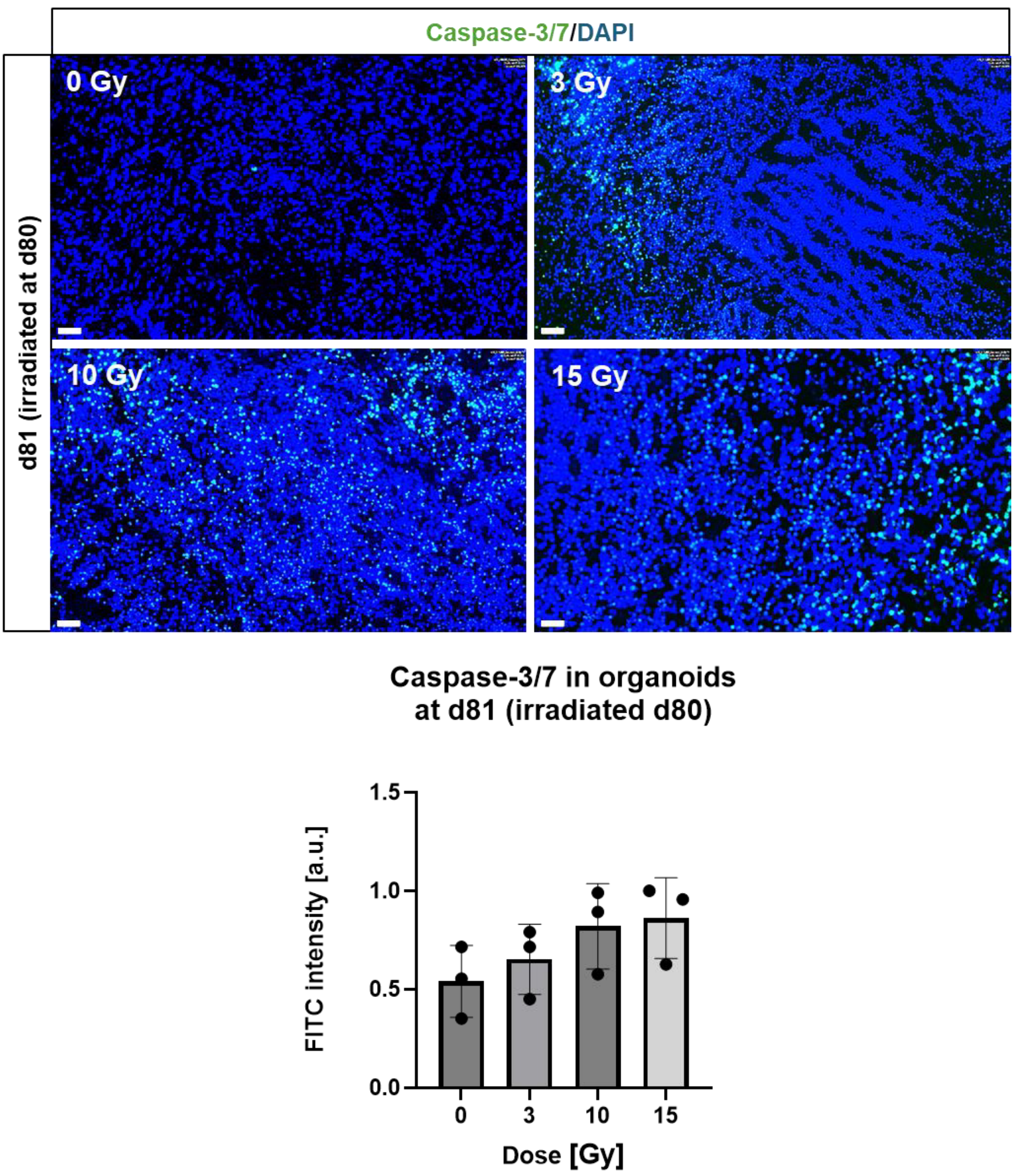
Caspase-3/7/DAPI
d81 (irradiated at d80)
0 Gy
3 Gy
10 Gy
15 Gy
Caspase-3/7 in organoids
at d81 (irradiated d80)
FITC intensity [a.u.]
1.5
1.0
0.5
0.0
0
3
10
15
Dose [Gy]

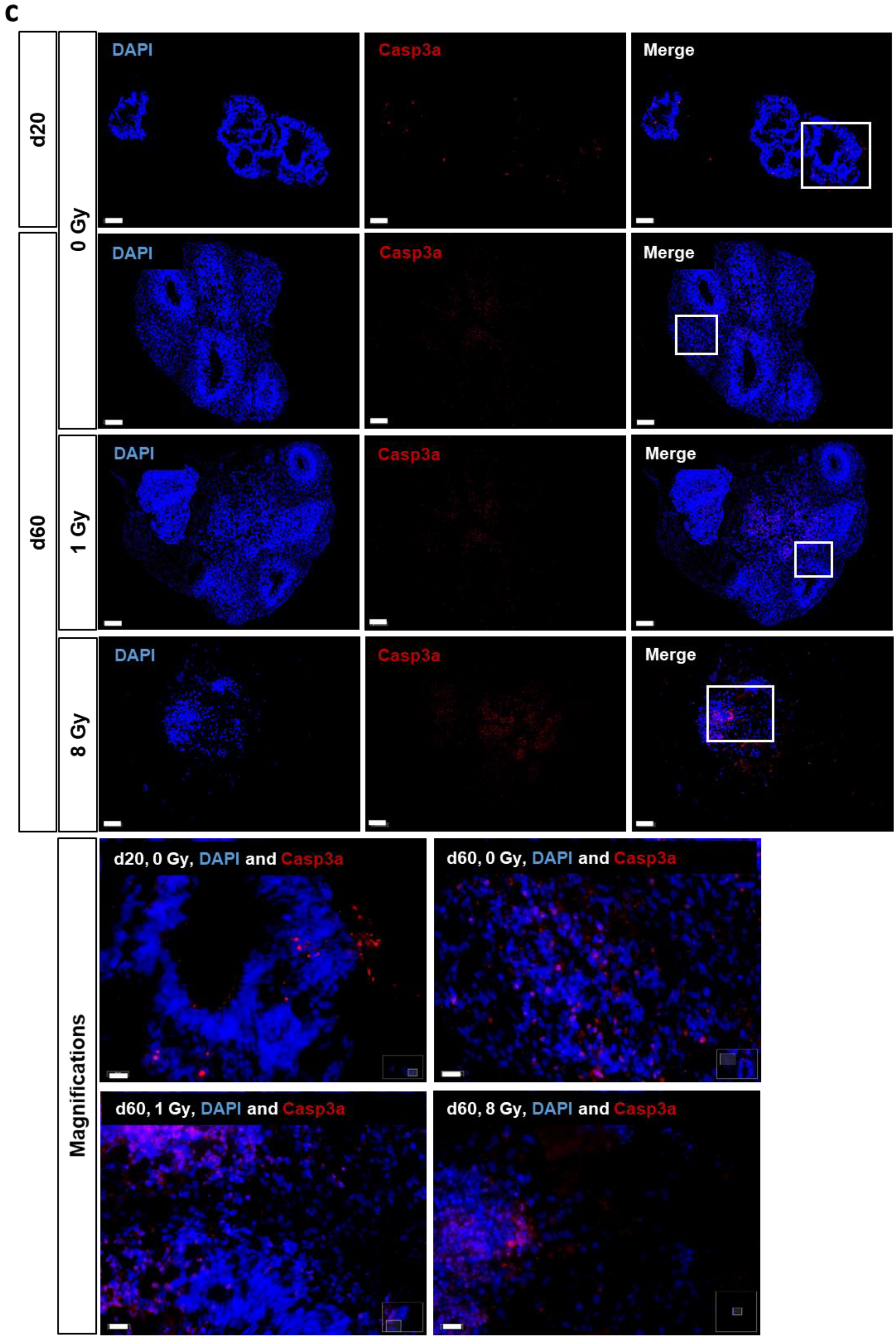
c
d20
d60
0 Gy
1 Gy
8 Gy
DAPI
Casp3a
Merge
Magnifications
d20, 0 Gy, DAPI and Casp3a
d60, 0 Gy, DAPI and Casp3a
d60, 1 Gy, DAPI and Casp3a
d60, 8 Gy, DAPI and Casp3a

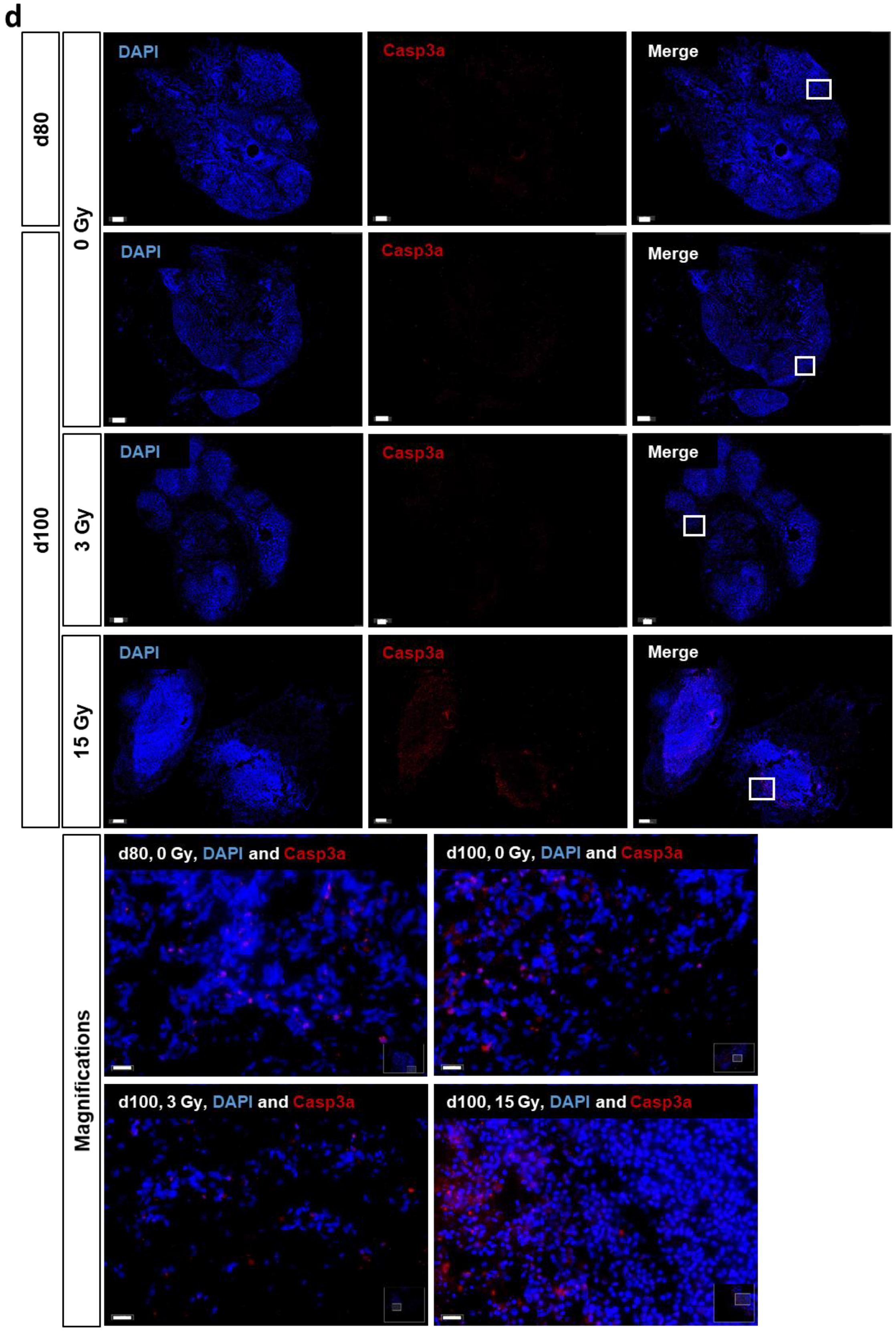

Figure S2. **Analysis of apoptosis in organoids. a-b** Representative immunofluorescence staining of Caspase-3/7 (green) in d21 and d81 cerebral organoids 24 h after irradiation with different doses of X-rays, nuclei stained with DAPI (blue), scale bar: 20 µm. **c-d** Representative immunofluorescence staining of active Caspase 3 (red) in control organoids at d20 and d80,

and in d60 and d100 cerebral organoids 40 or 20 days after irradiation with different doses of X-rays, nuclei stained with DAPI (blue), scale bar: 200 µm, and 20 µm for magnification.

Quantitative analysis of the dose-response curve for liquid-filling cavities

Data in Figure 3B and C were fitted by a linear function:

$$Y = \alpha D$$

where D is the dose in Gy, Y the percentage of organoids displaying cavities, and α (Gy^{-1}) the slope of the linear fit. For data at d20 (Figure 3B), the last data point was excluded, because clearly at high doses the curve does not grow linearly but tends to plateau. Linear fits were performed using the GraphPad Prism (v 9.3.1) software. For the data at d80 (Figure 3C) we also calculated the relative biological effectiveness (RBE) of the proton beam as:

$$RBE = \alpha_p/\alpha_X$$

where α_p and α_X are the slopes of the proton and X-rays dose-response curves, respectively. Uncertainty on the RBE is calculated by error propagation of the fitting parameter uncertainty. In clinical practice, a fixed RBE = 1.1 is used both for normal tissue (plateau region) and tumour (SOBP region)[1]. The result show that, for the endpoint of cavity formation, this clinical RBE is appropriate in the plateau but underestimates the proton effectiveness in the SOBP region. In clinical practice, protons are indeed considered more effective than X-rays at the same dose in inducing CEL in pediatric patients (see references[2],[3], and Discussion in the main text).

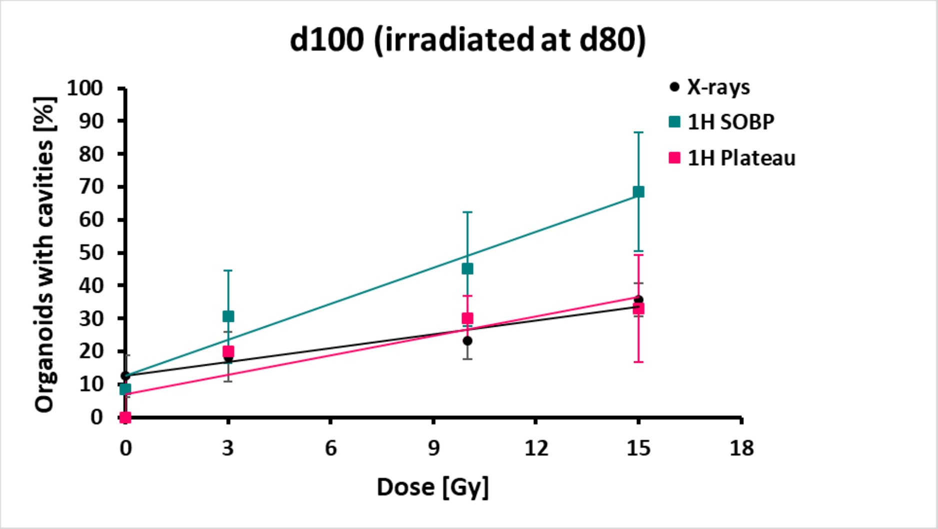

Figure S3. Percentage of organoids displaying cavities after irradiation at d80 (0 – 15 Gy) and with various radiation qualities: X-rays, protons in SOBP, and plateau, respectively including a linear fit of the data. Quantitative analysis of the dose-response curve and relative biological effectiveness are reported in Supplementary Table 1. Data are presented as mean ± SD for two to three independent experiments (N = 2-3) or as single experiment (N = 1) and n = 10 organoids per variant/group.

Supplementary Table 1. Quantitative analysis of the dose-response curve for the percentage of organoids with liquid-filling cavities

**Irradiation**	**α (Gy^{-1})**	**RBE**
X-rays, d20	25±3	-
X-rays, d80	1.9±0.4	-
Protons, plateau, d80	2.0±0.7	1.1±0.4
Protons, SOBP, d80	3.6±0.5	2.0±0.5

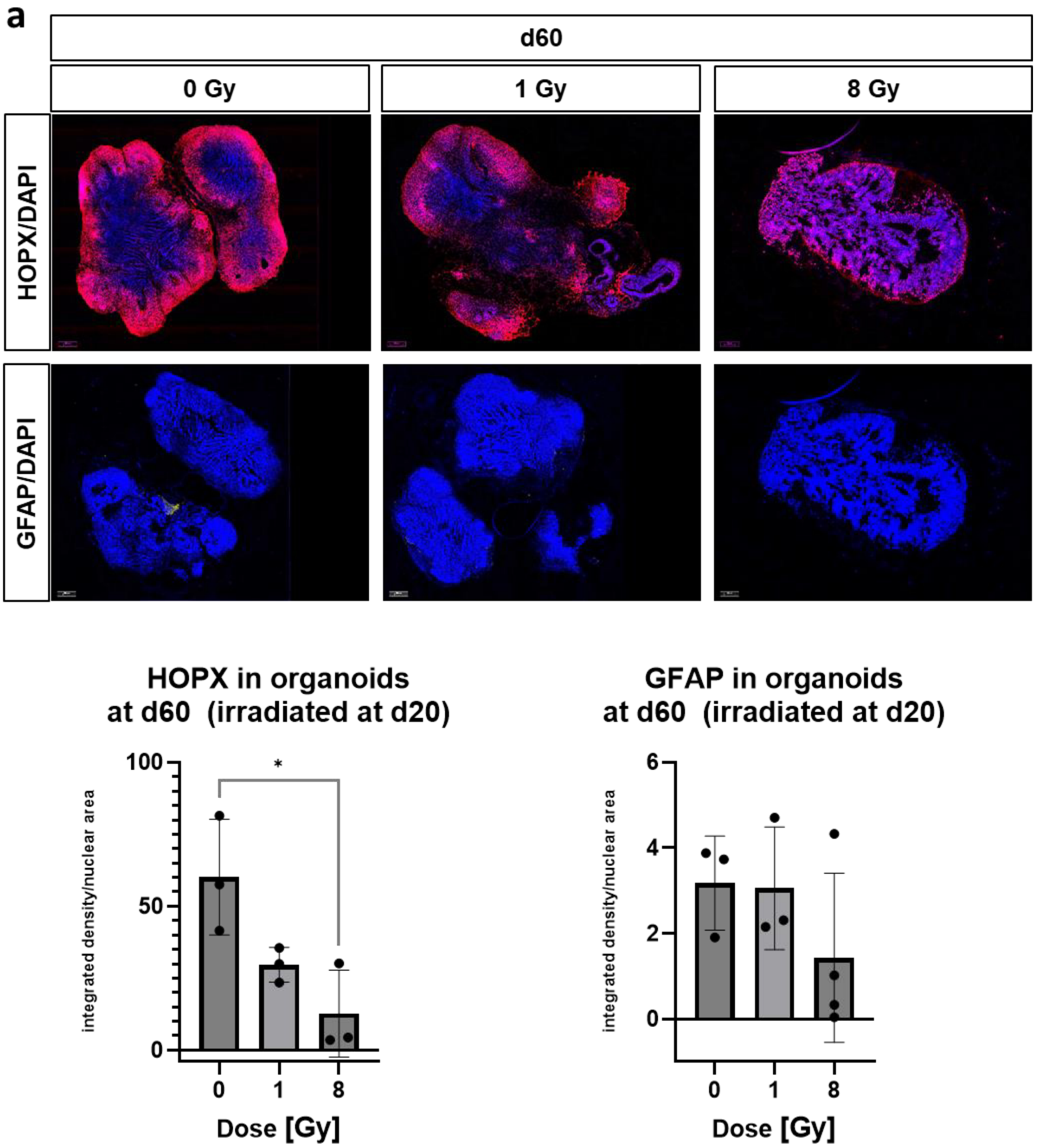
a
d60
0 Gy
1 Gy
8 Gy
HOPX/DAPI
GFAP/DAPI
HOPX in organoids
at d60 (irradiated at d20)
integrated density/nuclear area
100
50
0
*
0
1
8
Dose [Gy]
GFAP in organoids
at d60 (irradiated at d20)
integrated density/nuclear area
6
4
2
0
0
1
8
Dose [Gy]

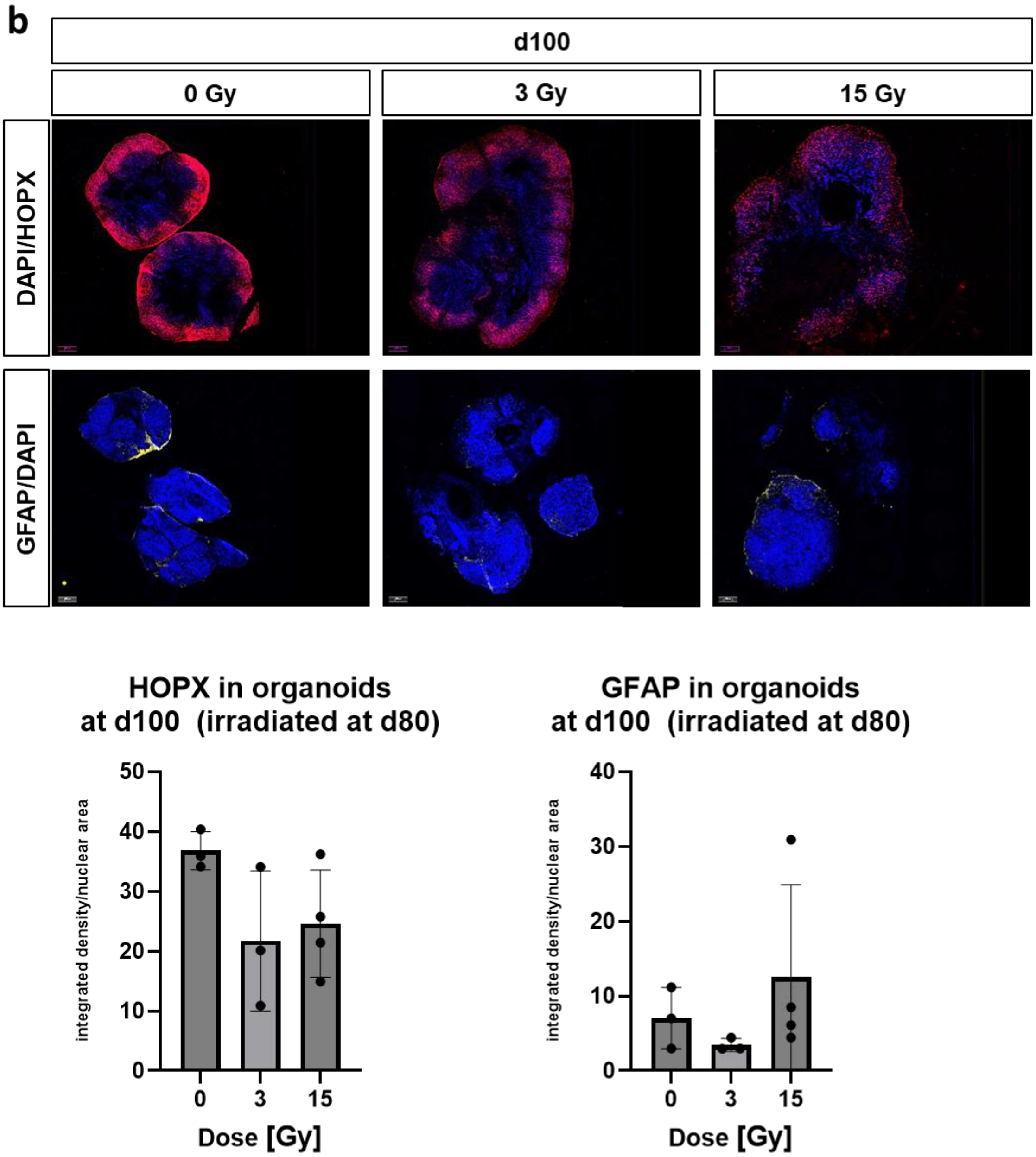

Figure S4. Expression of markers of outer radial glial cells, and astrocytes in irradiated organoids and their sham controls. a-b Representative immunofluorescence staining of HOPX (red) and GFAP (yellow) and their respective quantification at the protein level at d60 (X-ray irradiation at d20), and at d100 (X-ray irradiation at d80). Nuclei stained with DAPI (blue), scale bar: 100 µm. Data are presented as mean ± SD for three independent experiments (N = 3) and n = 1-2 organoid per experiment. Statistical analysis was done by one-way ANOVA with Dunnett´s post- test. * p = 0.0143; mean difference ± SE of difference: 47.47± 12.19; 95% CI of difference: 12.56 to 82.38.

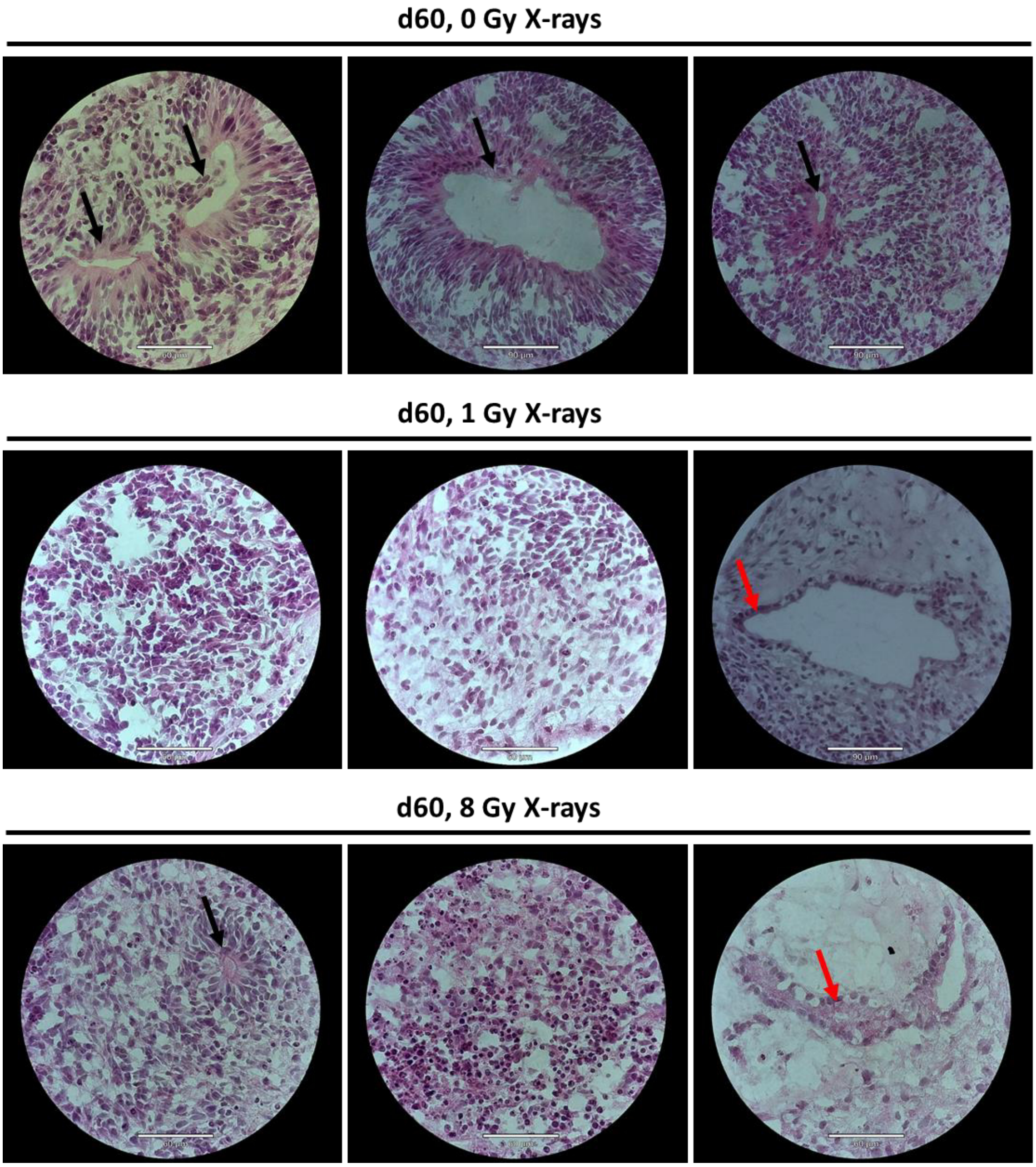

Figure S5. Monolayer of epithelial cells formed in d60 organoids subjected to 1 Gy or 8 Gy X-ray irradiation. Neuroepithelial proliferation zones (black arrows) and epithelial linings (red arrow) in H&E staining. Scale bar: 60 and 90 µm.

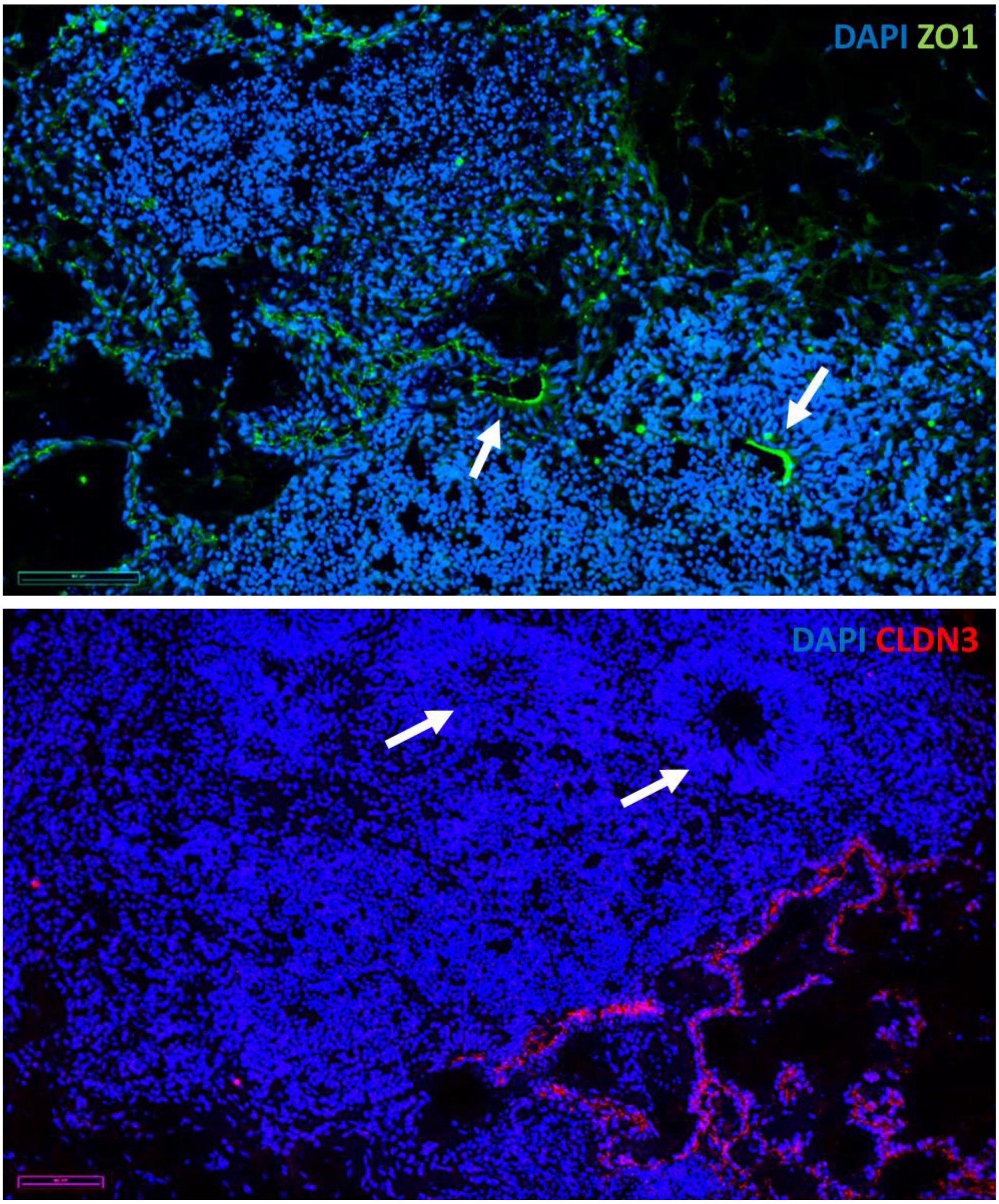

Figure S6. Choroid plexus (CP) formation in d66 cerebral organoids irradiated with 8 Gy X-rays at d20. Immunofluorescence stainings of the CP markers ZO1 (green) in neuroepithelial proliferation zones (white arrows) and in CP, and CLDN3 (red) in CP only. Nuclei stained with DAPI (blue), scale bar = 100 µm.

Supplementary Table 2. Primary Antibodies

Antigen	Species	Company	Catalog No.	Dilution	RRID
SOX2	rabbit	Thermo Fisher Scientific	A24339	1:100	AB_2924437
Nestin	mouse	BD Biosciences	611658	1:500	AB_399176
PAX6	rabbit	Abcam	ab195045	1:300	AB_2750924
Ki67	rabbit	Abcam	ab16667	1:500	AB_302459
Caspase 3 active	rabbit	R&D Systems	AF835	1:500	AB_2243952
ZO1	mouse	BD Biosciences	610966	1:125	AB_398279
CLDN3	rabbit	Sigma-Aldrich	HPA014361	1:400	AB_2668982
AQP1	rabbit	Sigma-Aldrich	HPA019206	1:500	AB_1844965

Supplementary Table 3. Secondary Antibodies

Host	Target	Fluorophore	Company	Catalog No.	Dilution	RRID
donkey	anti-mouse	AF488	Thermo Fisher Scientific	A24350	1:250	AB_2924437
donkey	anti-rabbit	AF594	Thermo Fisher Scientific	A24343	1:1000	
goat	anti-mouse	AF568	Thermo Fisher Scientific	A11004	1:1000	AB_2534072
goat	anti-rabbit	AF594	Thermo Fisher Scientific	A11012	1:1000	AB_2534079
goat	anti-mouse	AF647	Thermo Fisher Scientific	A21235	1:1000	AB_2535804
goat	anti-rabbit	AF647	abcam	ab150083	1:500	AB_2714032

Supplementary Table 4. Directly Labeled Antibodies

Antigen	Fluorophore	Species	Company	Catalog No.	Dilution	RRID
MAP2	AF647	rabbit	Abcam	ab225315	1:300	AB_3517252

Supplementary Table 5. Primers for qRT-PCR

Gene	Accession	Primer sequence (5´- 3´)
18s rRNA	NR_003286.2	ACTCAACACGGGAAACCTCACC (s)
		CGCTCCACCAACTAAGAACGG (as)
SOX2	NM_003106.4	CACTGCCCCTCTCACACATG (s)
		CCCATTTCCCTCGTTTTTCTT (as)
Nestin	NM_006617.1	CAGCTGGCGCACCTCAAGATG (s)
		AGGGAAGTTGGGCTCAGGACTGG (as)
PAX6	NM_000280.4	GAGCGGTGCATTTGCATGTT (s)
		TCTCAGATTCCTCTGTCATCATCC (as)
MAP2	NM_002374.3	TGCGCTGATTCTTCAGCTTG (s)
		TGTGTCGTGTTCTCAAAGGGT (as)
MSX1	NM_002448.3	CACTGAGACGCAGGTGAAGA (s)
		CCAGCTCTGCCTCTTGTAGT (as)
LMX1A	NM_177398.4	GGATGGTATTGTTCTGTGCTAGG (s)
		GTGTTCAAGTCTCCAATGATGTCC (as)
OTX2	NM_001270524.2	CCCTCACTCGCCACATCTAC (s)
		GGTTCAGAGTCCTTGGTGGG (as)
ZO1	NM_003257.4	CTCGCTCTCGGGAGATGTTT (s)
		CTCCATTGCTGTGCTCTTGG (as)
AQP1	NM_198098.4	TGGACACCTCCTGGCTATTG (s)
		GGGCCAGGATGAAGTCGTAG (as)
CLDN3	NM_001306.4	CCACGCGAGAAGAAGTACAC (s)
		CCTGCGTCTGTCCCTTAGAC (as)
IGF2	NM_000612.6	CGCTGTTCGGTTTGCGA (s)
		GATTCCCATTGGTGTCTGGAAG (as)
KIR7.1	AB013891	CCCACCTGAAAACCACACTACTG (s)
		GCATGAGGCCTAGGAGCATTTG (as)
NOTCH1	NM_017617.5	CTGGTCAGGGAAATC GTG (s)
		TGGGCAGTGGCAGATGTAG (as)
NOTCH2	NM_024408.4	TGTGACATAGCAGCCTCCAG (s)
		CAGGGGGCACTGACAGTAAT (as)
NGN2	NM_024019.4	CGCATCAAGAAGACCCGTAG (s)
		GTGAGTGCCCAGATGTAGTTGTG (as)
HES1	NM_005524.4	ATCCGGAGCTGGTGCTGATA (s)
		TTGGTGATCAGTAGCGCTGT (as)
HES5	NM_001010926.4	TGAAGCACAGCAAAGCCTTC (s)
		GAACTGCACGGCCTCCT (as)
WNT3	NM_030753.5	CCTCGCTGGCTACCCAATTT (s)
		GCTGGGCATGATCTCGATGT (as)
WNT5a	NM_003392.7	ATTCTTGGTGGTCGCTAGGT (s)
		TCCTTGAGAAAGTCCTGCCA (as)
LEF1	NM_016269.5	CCCATCACGGGTGGATTCAG (s)
		TGAGGCTTCACGTGCATTAGG (as)
BMP4	NM_001202.6	CGCTACTGCAGGGACCTATG (s)
		TCCATGATTCTTGACAGCCAAT (as)

References

1 Underwood, T. S. A. *et al.* A systematic review of clinical studies on variable proton Relative Biological Effectiveness (RBE). *Radiother Oncol* **175**, 79-92, doi:10.1016/j.radonc.2022.08.014 (2022).

2 Eulitz, J. *et al.* Increased relative biological effectiveness and periventricular radiosensitivity in proton therapy of glioma patients. *Radiother Oncol* **178**, 109422, doi:10.1016/j.radonc.2022.11.011 (2023).

3 Bahn, E. *et al.* Late Contrast Enhancing Brain Lesions in Proton-Treated Patients With Low-Grade Glioma: Clinical Evidence for Increased Periventricular Sensitivity and Variable RBE. *Int J Radiat Oncol Biol Phys* **107**, 571-578, doi:10.1016/j.ijrobp.2020.03.013 (2020).
